# Single-Port Laparoscopic Surgery in Children: Concept and Controversies of the New Technique

**DOI:** 10.1155/2012/232347

**Published:** 2012-06-12

**Authors:** Felix C. Blanco, Timothy D. Kane

**Affiliations:** ^1^Sheikh Zayed Institute for Pediatric Surgical Innovation, Children's National Medical Center, Washington, DC 20010, USA; ^2^The George Washington University School of Medicine and Health Sciences, Washington, DC 20052, USA; ^3^Department of Surgery, Children's National Medical Center, 111 Michigan Avenue Northwest, Washington, DC 20010, USA

## Abstract

Single-incision laparoscopic surgery (SILS) is emerging as an alternative technique to conventional laparoscopy for the treatment of common surgical diseases. Despite its wide use, the adoption of SILS in children has been slower since the broad application of minimally invasive techniques in children, in general, has historically lagged behind those in adults. This paper reviews the evolution of SILS from its original conception and its application in the field of pediatric surgery.

## 1. Introduction

The conception of laparoscopic surgery revolutionized the management of numerous surgical conditions and brought significant advantages over open surgery, beneficial for both the patient and the surgeon. Decreased postoperative pain, reduced operative times, faster recovery, and excellent cosmesis are now well-known attributes of minimal access surgery. 

Laparoscopy had constantly evolved with the intent to make surgery “scarless.” Two-port laparoscopic cholecystectomy, described by a group in Hong Kong in the late 90s, was perhaps the first sign of this new trend [1]. Without doubt, minimally invasive surgery is now inevitably moving towards even less invasive procedures which require a reduced number of access ports. 

Single-incision laparoscopic surgery (SILS) originated from the concept of natural orifice transluminal endoscopic surgery (NOTES), which emerged as an option to laparoscopy. The access to the peritoneal cavity through normal viscerae and the risk for intra-abdominal contamination was, however, a troublesome concern with NOTES. To address these issues, surgeons began to use the umbilical scar as the portal of entry to the abdomen, giving origin to “transumbilical surgery” or SILS. 

It was only a few years ago that SILS was applied to common surgical procedures, such as appendectomy and gastrostomy. Early reports of SILS describe the placement of multiple ports through a single incision with additional retraction utilizing transabdominal sutures. Retraction of the appendix with transabdominal “sling” sutures through the mesoappendix is an example of a commonly used strategy in the early stages of SILS appendectomy [2]. More recently, innovative techniques evolved into more complex laparoscopic procedures including nephrectomy, splenectomy, adrenalectomy, and bowel resection with intracorporeal anastomosis [3–6].

## 2. Single-Incision and Single-Port Laparoscopy 

In the beginning of the SILS era, the lack of proper devices to gain access to the peritoneal cavity motivated surgeons to implement new techniques and to generate innovative ideas. Home-made devices were initially used as an alternative to the currently available multichannel ports [7, 8]. An example of this was the use of a single-access device made of a surgical glove introduced through an umbilical incision; each finger of the glove was used to fit a separate laparoscopic instrument [9].

**Figure 1:**
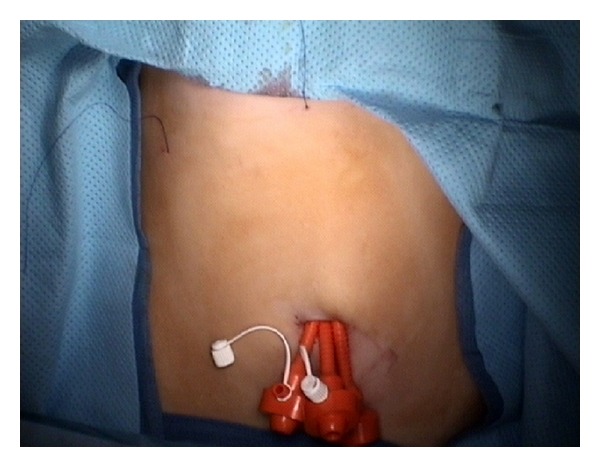
Single-incision multiple-trocar technique. Three low-profile trocars are inserted through separate contiguous incisions in the fascia. A transabdominal suture used to retract the gallbladder fundus is shown in the RUQ.

More recently, access to the abdomen was accomplished by introducing three 3–5 mm trocars through separate but contiguous incisions in the fascia under the same skin incision, a technique commonly used in small children (Figure 1). The separate fascial incisions are connected into a single incision at the end of the procedure to facilitate the extraction of the resected specimen. When the working space is limited, as is the case in neonates, accessory laparoscopic instruments are inserted directly through fascial stab wounds to avoid trocar crowding [10]. As expected, carbon dioxide leak can be significant with this technique [11]. 

The increasing need for an optimal access platform in SILS led to the invention of a multichannel “cannula” by a group in Spain [12]. The idea of introducing multiple instruments through a single device or port was well received by surgeons making possible the development of sophisticated ports for laparoscopic and thoracoscopic procedures [13–18]. Modern *access ports* can carry multiple trocars; these include the R-port, Uni-X Single Port, TriPort, and Quadport systems and allow the simultaneous introduction of multiple laparoscopic instruments and permit insufflation with an airtight seal. However, the large size of these devices (which may require a 2-3 cm fascial incision) often precludes the use in small children. 

Despite the development of improved single-access ports, the need for instrument triangulation remained a concern when using SILS. Our experience with standard straight laparoscopic instruments for cholecystectomy and other single-incision procedures was satisfactory; however, we observed that it requires expertise and demands longer operative times [10]. Hansen and colleagues emphasized the importance of using graspers of different lengths and upside-down grip of instruments to avoid instrument and hand clashing when working with straight conventional laparoscopic instruments [11]. Novel instruments with bent tips and roticulating mechanisms address, to some extent, this issue and have the benefit of avoiding in-line viewing and clashing of instruments [11, 19]. Unfortunately, the availability of these sophisticated instruments is restricted, its cost is high, and its applicability to young children is limited by their large size. 

Some surgeons routinely place a thin grasper (2 mm Minilap Alligator-Stryker Endoscopy, San Jose, CA) through the same or a remote fascial incision to assist with retraction [20]. A group in Argentina designed laparoscopic magnetic graspers that allow organ retraction when coupled with external magnets during SILS [21]. These magnets effectively provide retraction and overcome the lack of adequate triangulation. 

Harmonic scalpel and LigaSure (Covidien Norwalk, CT, USA) are coagulation/cutting devices commonly used in SILS. These devices seem to simplify the dissection of tissues and reduce operative times when comparing SILS to conventional laparoscopy in adults [22]. SIL splenectomy utilizing a combination of harmonic scalpel and LigaSure was safely performed in children [23].

Finally, as laparoscopic instruments evolve, newly developed angled light cord extensions and extralong endoscopes (>50 cm) allowed enhanced visualization and better maneuverability without interfering with the already hand-crowded single port [19]. 

## 3. SILS in Children

SILS was introduced in children much later than in adults [4, 7, 24]. This delay may be due to the perception that the small scars left by pediatric laparoscopic instruments were acceptable. Most likely, use of SILS in children has been slower since the broad application of minimally invasive techniques in children, in general, has historically lagged behind those in adults. Moreover, there is a concern regarding the limited maneuverability of laparoscopic instruments in the small peritoneal cavity of children, which is already challenging even with multiple trocar laparoscopy. 

In spite of these uncertainties, pediatric surgeons considered performing more complex procedures with less invasive techniques. Soon enough, single-port gastrostomy proved to be a suitable technique in children [24]. Later, Rothenberg and colleagues validated the use of SILS in the pediatric patient describing their experience on laparoscopic cholecystectomy. Their technique used an operating laparoscope, through which a single working instrument could be introduced. Often, they had to insert an additional instrument through a separate incision and use transabdominal sutures to retract the gallbladder [25]. 

**Figure 2:**
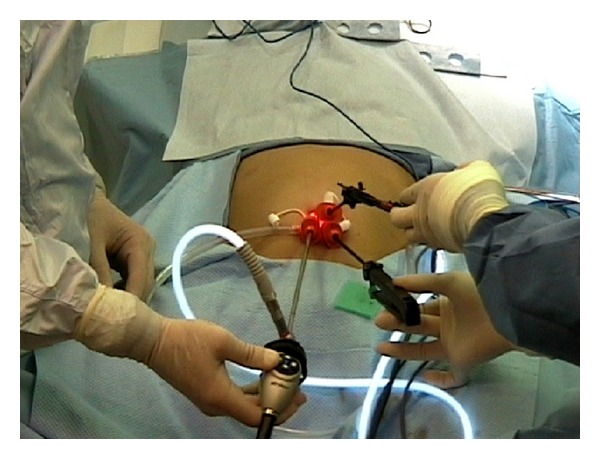
Multitrocar port inserted for single-incision laparoscopic cholecystectomy. An extralong endoscope and two instruments with different lengths were used to avoid hand clashing.

Although popular among adult SIL procedures, the use of multichannel ports is limited in small children due to their large size. Instead, many pediatric surgeons often prefer to place several 3–5 mm ports through a single umbilical wound, (Figure 1) as well as transabdominal sutures. These sutures are used to encircle the round ligament for liver retraction and often include seromuscular bites through the wall of various hollow organs including the gallbladder, stomach, or mesoappendix [2, 10, 11]. These “retracting” stitches are a common practice among pediatric surgeons and are particularly useful in small children due to their thin abdominal wall (Figure 2). 

An acceptable technique for retraction consists in the placement of thin graspers through remote stab incisions or through the same fascial opening [11]. 

## 4. Single-Incision Laparoscopic Appendectomy 

Two techniques of SIL appendectomy are currently available as follows.

### 4.1. Intracorporeal SIL Appendectomy

Intracorporeal SIL appendectomy is commonly performed with the three-trocar technique. Two 5 mm and one 3 mm low-profile trocars are introduced through separate fascial openings after a curvilinear infraumbilical incision is made in the skin. The trocars are generally positioned at 2, 6, and 10 o'clock position.

An angled 30° camera is introduced through one of the 5 mm ports and its tip kept close to the abdominal wall to avoid clashing with the working instruments. The appendix is retracted with a grasper and the mesoappendix followed to its base where it is divided with hook cautery. The appendix is then double ligated with endoloops, divided with scissors, and retrieved using one of the three following techniques: (1) direct removal through the umbilicus, (2) inserting the finger of a surgical glove and placing the specimen within this for retrieval, or (3) use of conventional endoscopic retrieval bag inserted alongside the camera and grasping instrument. To facilitate removal, the three small incisions are connected into one, and the wound closed in layers. 

### 4.2. Extracorporeal SIL Appendectomy

In this technique, a single 10 mm trocar is inserted through the umbilicus with a semiopen technique. A blunt grasper is introduced through the single channel of an operating laparoscope to mobilize the appendix from inflammatory adhesions until the mesoappendix is exposed. It is then grabbed, gently pulled inside the trocar, and removed simultaneously with the scope. Once exteriorized, the appendix is ligated and divided outside the abdomen with a standard technique. The appendiceal stump is then returned to the peritoneal cavity and the incision closed. 

## 5. Single-Incision Laparoscopic Cholecystectomy

SIL cholecystectomy (SILC) is one of the most popular procedures in both adults and children. Our technique of SILC includes the placement of an SILS port (Covidien, Norwalk, CT) in older children and the placement of three 5 mm ports through separate openings in the fascia with a technique similar to that of intracorporeal appendectomy. After the fascia is exposed, a Veress needle is introduced to achieve pneumoperitoneum.

In SILC, obtaining the critical view of safety to properly visualize the cystic duct and artery is perhaps of utmost importance. As mentioned previously, the limited instrument triangulation makes this task challenging, enforcing the use of additional ports. We often use transabdominal sutures to retract the gallbladder fundus or infundibulum and introduce a 2 mm Minilap Alligator grasper (Stryker Endoscopy, San Jose, CA, USA) through the umbilicus or a separate RUQ incision. Once the gallbladder is properly retracted, the cystic duct and artery are identified, double clipped, and divided. The gallbladder is then dissected off the liver bed with hook cautery and, when completely detached, it is extracted from the peritoneal cavity through the umbilical fascial defect, which is converted to a single incision of approximately 2 cm. The incision is closed with standard technique. If made, small incisions to fit 2 mm instruments are simply approximated with a single inverted subcuticular stitch.

Our initial experience with SILC had outcomes comparable to those of standard laparoscopy with no conversions to open cholecystectomy. Only seven percent of patients required at least one additional port [10].

## 6. Other SIL Procedures 

Many centers with modern laparoscopic capability rapidly expanded the indications of SILS. In children, SIL pyloromyotomy, splenectomy, nephrectomy, inguinal hernia, fundoplication, diaphragmatic hernia repair, and bowel surgery have been described [10, 11, 26, 27]. Tormenti and colleagues recently reported a technique of SILS ventriculoperitoneal shunt placement in children with hydrocephalus [28]. The direct visualization of the shunt as it enters the peritoneal cavity and the avoidance of an abdominal incision contiguous to the shunt are attractive attributes of this novel technique. 

Procedures not fully developed in children but available for adults include adrenalectomy, liver resections, colectomy with intracorporeal anastomosis, and single-incision thoracoscopy [18, 29–31].

## 7. Outcomes of SILS 

Without doubt, the cosmetic appearance of a literally “scarless” procedure is one of the greatest attributes of SILS. The use of the umbilical scar as the single portal of entry for the instruments allows for a more conventional and safe option compared to NOTES. Yet, this cosmetic advantage may not be as relevant in children who usually outgrow the size of the routine 3 and 5 mm incisions used in conventional laparoscopy. As an additional benefit, the umbilical incision can, as it routinely is, be used for specimen retrieval and converted to a circumumbilical incision when there is need for a larger incision. 

Despite the limited number of incisions, no major differences exist in the recovery time or need for postoperative analgesia when SILS is compared to conventional laparoscopy. The postoperative length of stay after cholecystectomy was similar for children undergoing either technique in one series [32]. A recent randomized controlled trial showed that patients who underwent SIL cholecystectomy experienced less postoperative pain and required fewer analgesics compared to those who were treated with conventional laparoscopic cholecystectomy [33]. In spite of the encouraging outcomes of SILS [34], level 1 evidence showed that SIL appendectomy was associated with increased requirement of analgesics, longer operative times, and higher hospital charges compared to the standard approach [35]. 

Unfortunately, the need for specialized laparoscopic equipment reduces the cost-effectiveness of SILS. Though feasible in experienced hands, use of conventional laparoscopic instruments in SILS prolongs the operative times and makes the learning curve steeper. As the operative times are reduced with the utilization of specially designed equipment, this negatively affects the overall cost of surgery. We believe that longer operative times can be significantly reduced as experience is gained by the operating surgeon and with the use of roticulating instruments [36, 37]. The limited availability and high cost of angled graspers and multichannel ports significantly increase the operative costs, as we mentioned before.

Reported intraoperative SILS complications include bowel perforation, thermal injury, and bleeding [11]. In a series of 32 SIL pyloromyotomies, the reported complication rate was 6% including duodenal and pyloric mucosal perforations [11].

Ponsky and colleagues published their experience with more than 70 pediatric SILS cases including cholecystectomy, appendectomy, and gastrostomy. They reported an acceptable rate of conversion to conventional laparoscopy and a low incidence of postoperative complications [22]. In other series including adults and children, the outcomes of SILC were comparable to standard laparoscopic cholecystectomy with no major postoperative complications and a conversion rate of 2 to 11% [10, 38–40]. Conversion to standard laparoscopy or the addition of extra ports should not be considered a complication of SILS. Under no circumstances should the surgeon compromise patient safety and utilize sound judgment when considering adding extra ports or retraction stitches, when necessary. 

Recent reports indicate that elective SILS cholecystectomy is safe when done in the outpatient setting.

## 8. The Future of SILS in Children

The development of sophisticated laparoscopic instruments with multidirectional roticulating and articulating capabilities will soon allow the pediatric surgeon perform complex laparoscopic procedures in a more efficient and easy way. With these, limited triangulation and tissue handling will no longer be an issue. In addition, the development of smaller, low-profile SILS ports will ease the maneuverability of laparoscopic instruments and avoid trocar crowding in the already reduced operative field of children. 

In spite of the early reported success of SILS, we believe that there are still formidable obstacles which must be overcome in order to optimize this approach in children. Certainly, the boundless creativity of the surgeon in search for less invasive methods of performing operations may eventually evolve into the ideal “scarless” surgery.
